# Chlorate of Potassa in Scrofulous Cases

**Published:** 1873-03

**Authors:** C. A. Clarkson

**Affiliations:** Marshall, Missouri


### SELECTIONS.

---

### CHLORATE OF POTASSA IN SCROFULOUS CASES.

BY DR. C. A. CLARKSON, MARSHALL, MISSOURI.

Early in the fall of 1870 I was called to see a female mulatto child, aged about two years, and of a scrofulous diathesis. I found her feverish from dentition, with considerable diarrhœa. Prescribed small dose of the mild chloride of mercury and Dover's powders, with syrup of ipecac. On the following day, the fever had subsided and the diarrhœa abated, the mercurial having acted finely, and the child was apparently as well as usual.

About ten days after, I was again called to see the child. Found her in much the same condition as before, but with aggravated symptoms; fever high, tongue coated, diarrhœa present, tenderness over the right hypochondriac region, and the abdomen slightly swelled. I prescribed calomel and Dover's powder in doses of one grain each every four hours until five doses were taken, with syrup of ipecac. On the next day the patient was but little if any better, though the mercury was acting freely upon the liver, and the diarrhœa had in a great measure disappeared. She was very feverish and fretful, tongue foul, with a brownish coat—tenderness over the liver not so great, but abdomen more swelled and very tender to the touch. Ordered the Dover's powders continued, oil to be given in twelve hours, and a blister to be applied over the right hypochondriac region. On the following day, the blister having drawn well, most of the symptoms had subsided; no diarrhœa, very little fever, tongue cleaning, abdomen less swelled and less tender, but the child looked very languid and feeble. Prescribed one grain of sulphate of quinine, and two grains of Dover's powder, twice a day.

Two days later, the symptoms as detailed above had pretty much all disappeared; but some one of them, or all of them together, had evidently stimulated the scrofulous taint in the child's blood into vigorous action, and it had already made considerable headway in its ravages. The eyelids were swelled and puffy, conjunctiva inflamed, throat swelled both inside and out, and abdomen still tympanitic. Tumors were evident about the neck, axilla and groins. There was no fever, or very little; tongue nearly clean, and the blister almost healed. Ordered a full dose of castor oil; quinine, one grain; muriated tincture of iron, three drops, three times a day, and Dover's powder, two grains at night.

One week later I found my patient with all the scrofulous symptoms fearfully aggravated. Throat, neck and face swelled enormously, eyes almost closed, and the other features hardly distinguishable, and many of the glandular tumors were suppurating. The languor was extreme, and the complexion (she was a very bright mulatto) had the dead white appearance common to chlorosis. Continued quinia, and substituted iodide of iron for the muriated tincture. Prescribed also, as a lotion, tincture of iodine, two drachms; iodide of potassium, four drachms; alcohol, two ounces; distilled water, five ounces. With this the tumors and ulcerations were to be washed daily, and a saline laxative given daily, to keep the bowels open.

A week later I again saw the child. Worse in every respect. Tongue dry, foul, and very dark; ulcerations everywhere; throat, gums, mouth, face, neck, axillas, groins, thighs, and even legs, a mass of festering putridity horrible to look at, and horrible to smell, too—the ulcerations discharging a sero-purulent fluid that was extremely feted. The discharges from the bowels now were also frequent, with an odor and an appearance curiously similar to that from the external ulcerations. I prescribed iodide of potassium and tincture of opium, in such doses as could be borne, to be steadily continued. Directed carbolic acid as a deodorizer and as a lotion, and continued quinine, with whisky.

In ten days I returned, and though I found the poor creature still alive, could discover no trace of improvement. She was fearfully emaciated. The gums were black and ulcerating, the tongue swelled and dark. On my last visit I had informed the mother that there was no hope for her child, and I was really surprised to find it alive. It seemed hopeless and almost cruel to worry the suffering thing with medicine, other than an anodyne to mitigate the pain.

However, I resolved to make one more effort. Prescribing morphia to moderate the suffering as much as possible, I directed two drachms of chlorate of potassa to be dissolved in four ounces of water, and one teaspoonful to be given every three or four hours, and this, with the morphia, to be continued until the child died, or I returned.

In about a week I again visited the little patient, and, to my great astonishment, there was a marked *improvement* in her condition. Her treatment was, of course, continued, still using carbolic acid as a lotion and disinfectant, lessening the doses of potassa and morphia as convalescence became more and more decided. In five to six weeks the child was almost entirely restored to health, and is to-day as fat and healthy-looking as any child you will commonly see.

The recovery seemed little short of miraculous! What was the cause of it? Was it *post hoc* or *propter hoc*, as to the chlorate of potassa? These are questions I should like to see answered or discussed by some of the older and more experienced members of the profession. The rationale of my prescription was the known effect of chlorate of potash upon the red corpuscles of the blood, and my opinion that, in some way, it has the effect of lessening the fibrin of the blood. In scrofulous blood there is an abnormal deficiency of red corpuscles and excess of fibrin. Did the chlorate of potassa increase the one and decrease the other, and thus restore the healthy equilibrium? I have often used the chlorate *internally* to relieve ptyalism, and always with success; and it was this experience that suggested its use to me in this case, with the fact that I had read in some periodical, I do not remember which, the statement that chlorate of potash will, in some unknown way, dissolve the fibrin of the blood. I have used it, and always with happy effect, in the treatment of chlorosis; but its use in the treatment of scrofulous cases was new—at least was new to me. If the successful use of chlorate of potassa as a remedial agent in grave scrofulous cases is remarkable to the profession generally, as it certainly was to me, then the report of this case is of sufficient importance to find a place in your journal.—*Kansas City Medical Journal.*

